# Alpelisib Efficacy in Hormone Receptor-Positive HER2-Negative PIK3CA-Mutant Advanced Breast Cancer Post-Everolimus Treatment

**DOI:** 10.3390/genes13101763

**Published:** 2022-09-29

**Authors:** Ari Raphael, Mali Salmon-Divon, Jessica Epstein, Tamar Zahavi, Amir Sonnenblick, Shlomit S. Shachar

**Affiliations:** 1 Division of Oncology, Tel Aviv Sourasky Medical Center, Tel Aviv 642390, Israel; 2 Sackler School of Medicine, Tel Aviv University, Tel Aviv 69978, Israel; 3 Department of Molecular Biology, Ariel University, Ariel 4077625, Israel; 4 Adelson School of Medicine, Ariel University, Ariel 4077625, Israel

**Keywords:** metastatic breast cancer, everolimus, alpelisib

## Abstract

This real-world cohort analysis assessed the efficacy of alpelisib and endocrine treatment (ET) combinations in a post-everolimus setting. Thirteen women who started alpelisib and ET at standard doses between 2018 and 2022 for advanced breast cancer (ABC), after undergoing CDK4/6i and everolimus treatment, were eligible for the study entry. The primary endpoint was progression-free survival (PFS), and the secondary endpoints were the objective response rate (ORR) and clinical benefit rate (CBR), with different molecular profiling. The patients had previously received a median of four (range 3–8) systemic treatments, including CDK4/6i and everolimus. The median PFS on alpelisib was 5.5 months (range 0.5–10), and four women each had an ORR and three (23%) had a stable disease. The 6-month CBR was 46.1%, similar to the BYLeive study cohort C (47.8%). Notably, our cohort included patients with a long CBR under everolimus treatment (median 6 months, range 1–18); however, the responses to alpelisib and everolimus were not correlated (Pearson r = −0.23, *p* = 0.44). The *PIK3CA*, *P53*, *ARID*, *GATA3*, and *ESR1* mutations were not associated with the 6-month CBR. Despite heavy pre-treatments, including everolimus, alpelisib was clinically relevant in our cohort, even among patients with an *ESR1* mutation. The best treatment sequence for PIK3CA/mTOR inhibitors warrants examination in future trials on PIK3CA-mutant inpatients with luminal ABC.

## 1. Introduction

Breast cancer (BC) is the most common cancer diagnosis and the leading cause of cancer death among females worldwide [1]. The most common BC is the hormone receptor-positive (HR+) type. There has been major progress in the treatment of HR+ metastatic BC (MBC) over the past few years. The first line of treatment has been improved thanks to the combination of CDK4/6 inhibitors (CDK4/6i) and endocrine therapy (ET), which has enhanced both progression-free survival (PFS) and overall survival (OS) [2]. However, drug resistance, which develops in nearly all patients, remains a critical unmet medical need, and the optimal sequence of treatment after CDK4/6i remains unknown. There are few FDA-approved drugs for HR+ MBC, after the first-line for PI3K-mutated tumors. Until recently, everolimus, in combination with exemestane, has been given as a second-line treatment, since signaling through the mTOR pathway has been identified as a mechanism of resistance to ET in MBC [3]. Several studies have suggested that there is significant crosstalk between the estrogen-receptor (ER) pathway and the mTOR pathway [4]. The BOLERO-2 phase III trial demonstrated a significant improvement in PFS (7.8 months vs. 3.2 months, respectively, *p* < 0.0001), and in response rates (9.5% vs. 0.4%, *p* < 0.001) in women who had been given the combination everolimus+AI treatment, compared to exemestane alone. A very small number of studies showed a modest benefit of the combination of AI and everolimus-tested post-CDK4/6 therapy, demonstrating a PFS of ~4 months [5]. In 2019, alpelisib was approved by the FDA as a treatment for patients with MBC with PIK3CA mutations (~40% of HR+), based upon the SOLAR-1 phase III, randomized, double-blind, and placebo-controlled trial of alpelisib plus fulvestrant, versus the placebo plus fulvestrant. That trial included 572 patients with HR+ HER2-negative MBC, whose disease had progressed after the women received an AI. The mean PFS was longer with the combination in the group that had a *PIK3CA* mutation (11.0 months), compared to the placebo plus fulvestrant arm (5.7 months) (hazard ratio 0.65; 95% confidence interval (CI): 0.50, 0.85; *p* = 0.001) [6]. Given that both treatments are approved for HR+ MBC after progression on ET, and that both demonstrate a significant improvement in PFS, the optimal sequencing of the drug delivery emerges as a major gap in knowledge. The goal of this study is to contribute novel information towards filling that gap.

Based upon the above findings in the literature, we hypothesized that everolimus and alpelisib are not equivalent, and, since studies comparing everolimus to alpelisib or their sequential treatment are not expected, we therefore retrospectively analyzed the data from a cohort of women who had received alpelisib post-everolimus, in order to examine their PFS and response to alpelisib post-everolimus, and to explore if the response is associated with various biomarkers.

## 2. Methods

This institution-based, retrospective cohort study included data from the database of the Tel Aviv Sourasky Medical Center, Tel Aviv, Israel. The study received ethics committee approval by the institutional review board (0611-21-TLV). The eligible participants were patients diagnosed with MBC who were ER- and/or progesterone receptor (PR)-positive and HER2-negative, and who received everolimus and alpelisib during treatment for metastatic disease. The retrieved data on the demographic, clinical, and pathological parameters included the age, molecular analysis of the tumor, lines of therapy, and response to therapies. The patients’ clinical care was managed by the treating physician, based on the international guidelines.

### Statistical Analysis

The descriptive statistics are presented as the number (*n*), median, and range. The associations between the response rate, or the clinical benefit and genomic changes, were estimated with Fisher’s exact test. The OncoPrint figures and PFS survival curves were generated by means of the ComplexHeatmap and survfit R packages, respectively.

## 3. Results

Thirteen patients who were treated with alpelisib, following treatment with everolimus, were included in the analysis (see detailed individual treatment for each patient in the Supplementary Table S1), and next-generation sequencing NGS was carried out during the treatment course for metastatic disease in eleven of them (see test details in the Supplementary Table S2). The 13 patients’ characteristics are shown in Table 1. The cohort’s median age was 68 years (range 49–81). The median time from the metastatic diagnosis to alpelisib treatment was 3.5 years (1.4–11.6), and all 13 patients had been treated with CDK 4/6 before receiving the alpelisib.

### 3.1. Response to Everolimus and Alpelisib

**Figure 1:**
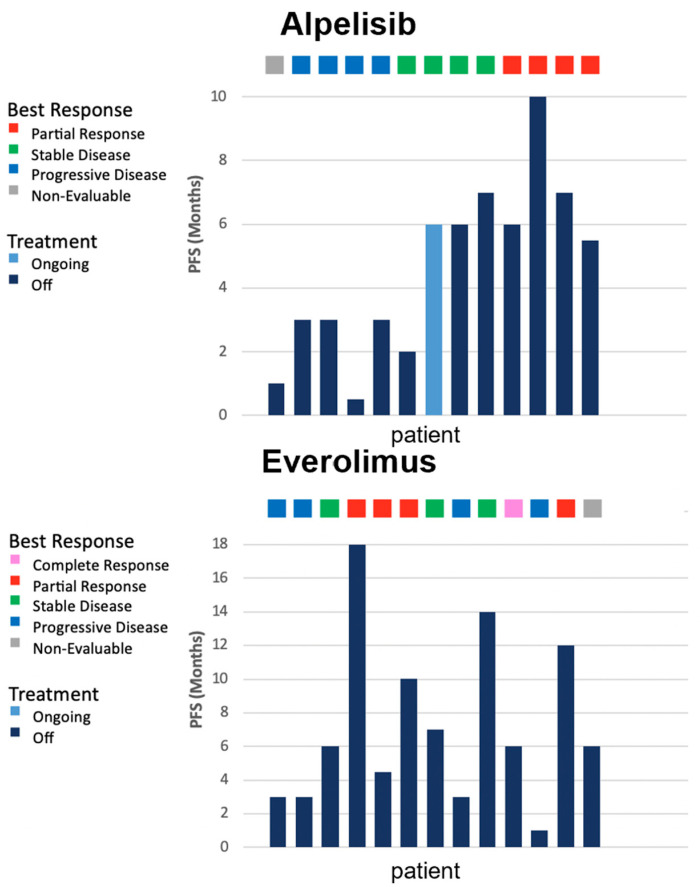
Progression-free survival and best response to everolimus and alpelisib. The same patient is represented in both graphs in the same bar location.

**Figure 2:**
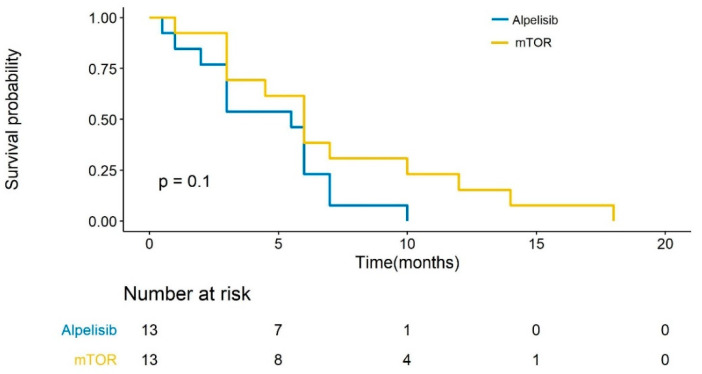
Kaplan–Meier curve—PFS with everolimus and alpelisib treatment.

Figure 1 displays the PFS and best response to each drug for each patient. Four of the thirteen patients who received alpelisib post-everolimus had a partial response; four had a progressive disease; and four had a stable disease. The median PFS on the everolimus was 6 months (range 1–18) and the median PFS on alpelisib was 5.25 months (range 0.5–10). No correlation was detected between the PFS of the drugs (Pearson correlation r = −0.23, *p* = 0.44). Kaplan–Meier curves for the progression-free survival probability for everolimus and alpelisib are presented in Figure 2, showing no significant difference in PFS.

### 3.2. Clinical Benefit of Alpelisib by Biomarkers

**Figure 3:**
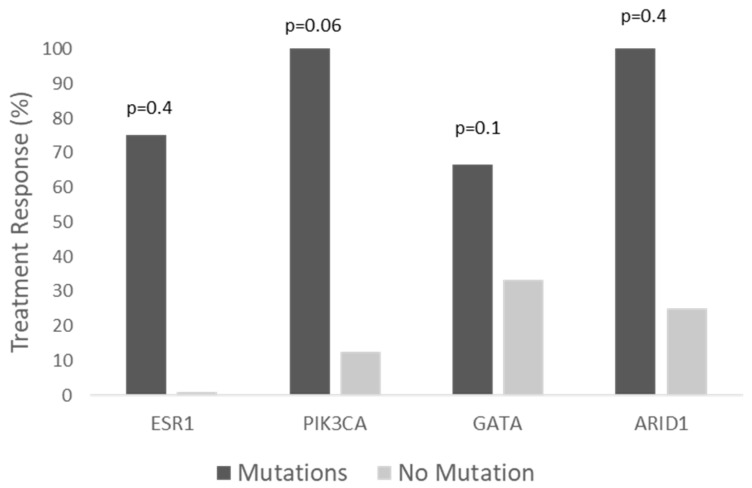
Treatment response by mutations. For PIK3CA, “Mutations” group represents double mutation, and the second group represents a single mutation.

**Figure 4:**
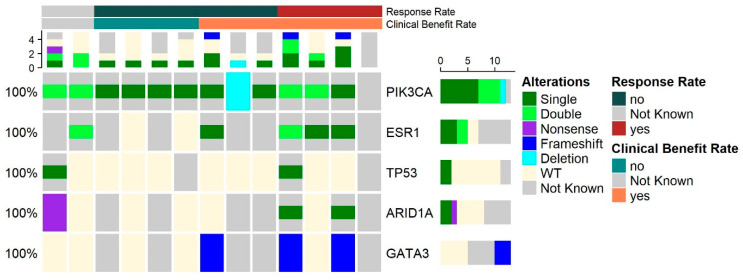
Genomic modifiers of response and outcome by treatment duration. OncoPrint of the dichotomous clinical benefit and response groups for 13 patients with broad profiling data (left: no benefit [*n* = 4, biologically independent samples], right: clinical benefit [*n* = 7, biologically independent samples]). From top to bottom: *PIK3CA*, *ESR1*, *TP53*, *ARID*, and *GATA3*, none of which significantly correlated with response rate or clinical benefit rate.

For the patients for whom the data on the NGS and response assessment were available, we performed an analysis by biomarker, which showed a clinical benefit regardless of the different mutations. The patients with ESR1 mutations had numerically different clinical benefits (including partial response and stable disease) from those with wildtype (WD) ESR1 (*n* = 6, 100% vs. 0%, respectively, *p* = 0.066), and the patients with PIK3CA double mutations had a numerically higher clinical benefit rate compared with the patients with a PIK3CA single mutation (*n* = 10, 100% vs. 50%, *p* = 0.46); those with GATA3 mutations had numerically higher clinical benefit rates (*n* = 6, 100% vs. 33%, *p* = 0.4); and those with ARID mutations had numerically higher clinical benefit rates compared with WT (*n* = 6, 100% vs. 50%, *p* = 0.46.) Looking at the objective response rate of the alpelisib treatment, only the PIK3CA showed a close-to-significant effect, indicating that the patients with double mutations had higher response rates than those with a single PIK3CA mutation (n = 10, 100% vs. 12.5%, *p* = 0.06), as demonstrated in Figure 3 and Figure 4.

## 4. Discussion

Alpelisib is the only *PIK3CA* inhibitor to have been approved for the treatment of HR+ MBC to date. Both everolimus and alpelisib are active in MBC and work on the same *PI3K/AKT/mTOR* axis, but the ideal sequencing to minimize treatment resistance is unknown, and even the new ESMO guidelines cannot recommend the best sequencing beyond the first line [7]. The PFS rates in the advanced treatment lines usually shorten from line-to-line of treatment [8].

In the BYLieve study, the percentage of patients alive and without disease progression at six months was 50.4% in the cohort A (patients who had progressed on CDK4/6 and ET and received alpelisib with fulvestrant), while the percentage of patients alive and without disease progression at six months was 48.7% in the cohort C (patients who were also treated with chemotherapy before alpelisib) [9]. Most of the patients in our study received alpelisib after four or more lines of treatment that included everolimus, and the six months without disease progression was 46.1%, which was very similar to both cohorts of the BYlieve study. This finding indicates that treatment with alpelisib post-everolimus can provide a clinical benefit in selected patients.

ESR1 mutations are associated with resistance to hormonal treatment [8]. In addition, Razavi et al. [10] reported, among the patients with HR+ MBC who participated in a phase I/II study of alpelisib in combination with letrozole or exemestane, that ESR1-activating mutations increased during treatment, and were associated with a resistance to the combination. Further support to this observation was demonstrated in the BYLeive trial, where patients with the ESR1 mutation tested in ctDNA had numerically but not significantly shorter PFS (6.3 vs. 8.3 months, *p* = 0.095) [11]. By contrast, in our study the patients with an ESR1 mutation had numerically but not significantly longer PFS than the patients with a WT ESR1 (mean of 6.1 vs. 2.3 months, *p* = 0.07). While this may reflect the lower numbers in our cohort, it also highlights that ESR1 mutations, per se, should not exclude patients from further hormonal and alpelisib treatment. We also found that the patients with more than one PIK3CA mutation had borderline more responses than the patients with a single mutation, as had been observed by others [12].

In an evaluation of the impact of PIK3CA hotspot mutations in cfDNA on everolimus efficacy among the BOLERO-2 study participants (*n* = 550), the median PFS in the everolimus vs. placebo arms was similar among patients with tumors that had either wild-type or mutant PIK3CA (HR 0.43 and 0.37, respectively). Everolimus also prolonged the median PFS in patients with PIK3CA H1047R and E545K/E542K mutations to a similar degree [13]. This suggests that, while patients with PIK3CA mutations clearly benefit from alpelisib, they may have the same benefit from everolimus, and therefore using both drugs in sequence is a relevant option for some patients [14].

Our study has several limitations that bear mention. It is retrospective in design and includes a small number of patients. Larger studies investigating the clinical benefit of sequencing these drugs are needed.

## 5. Conclusions

Our study findings demonstrate that alpelisib has clinical activity, post-everolimus treatment in a real-world cohort of heavily pretreated patients. They support the use of alpelisib in patients who have been exposed to other treatments in the same PIK3CA/AKT/mTOR pathway. Further studies with larger numbers of patients are necessary to validate the hypothesis that alpelisib treatment post-everolimus can be beneficial to patients with MBC.

## Figures and Tables

**Table 1:** Patients’ Characteristics (*n* = 13).

Characteristic	
Age, years, median (range)	68 (49–81)
Previous systemic treatment lines, *n*	
3	3 (23%)
4	5 (31%)
≥5	5 (38.5%)
Received chemotherapy before alpelisib	4 (31%)
Median time from metastasis to alpelisib treatment, years (range)	3.5 (1.4–11.6)

## Supplementary Materials

The following supporting information can be downloaded at: https://www.mdpi.com/article/10.3390/genes13101763/s1, Table S1: Treatment lines for the individual patients; Table S2: Detailed NGS test.
