# Supplementary material for: Alpelisib Efficacy in Hormone Receptor-Positive HER2-Negative PIK3CA-Mutant Advanced Breast Cancer Post-Everolimus Treatment
Source: Genes (Basel). 2022 Sep 29;13(10):1763. doi: 10.3390/genes13101763 (PMC9601363; doi:10.3390/genes13101763)
Supplement: Supplementary file 1 [file genes-13-01763-s001.zip › genes-1868966-supplementary.pdf]

Table S1A- treatment lines for the individual patients

Patient #	1st line	2nd line	3rd line	4th line	5th line
1	Palbociclib+Letrozole	**Affinitor+Fulvestrant**	Bevacizumab+Capecitabine		NA
2	Palbociclib+Letrozole	Fulvestrant	**Affinitor+Exemestane**	**Alpelisib+Fulvestrant**	NA
3	Palbociclib+Fulvestrant	**Affinitor+Exemestane**	**Alpelisib+Exemestane**	NA	NA
4	Palbociclib+Letrozole	Elacestrant	**Affinitor+Exemestane**	**Alpelisib+Fulvestrant**	Paclitaxel+Carboplatin
5	Letrozole	Palbociclib+Fulvestrant	**Affinitor+Exemestane**	**Alpelisib+Fulvestrant**	Navelbine PO
6	Tamoxifen	Palbociclib+Letrozole	abemaciclib+atezolizumab+Fulvestrant	**Affinitor+Exemestane**	**Alpelisib+Exemestane**
7	Palbociclib+Letrozole	Palbociclib+Fulvestrant	Capecitabine	**Affinitor+Exemestane**	Vinorelbine
8	Palbociclib+Letrozole	Fulvestrant	**Affinitor+Exemestane**	**Alpelisib+Fulvestrant**	NA
9	Palbociclib+Fulvestrant	**Affinitor+Letrozole**	**Alpelisib+Fulvestrant**	Paclitaxel	NA
10	Palbociclib+Letrozole	Fulvestrant	**Affinitor+Exemestane**	Capecitabine	Doxil
11	Palbociclib+Fulvestrant	**Affinitor+Exemestane**	**Alpelisib+Exemestane**	NA	NA
12	Palbociclib+Fulvestrant	**Affinitor+Exemestane**	Paclitaxel	Capecitabine	Vinorelbine
13	Avastin+Paclitaxel	**Affinitor+Exemestane**	Capecitabine	Palbociclib+Fulvestrant	Doxil

Patient #	6th line	7th line	8th line
1	NA	NA	NA
2	NA	NA	NA
3	NA	NA	NA
4	Capecitabine	NA	NA
5	NA	NA	NA
6	Capecitabine	NA	NA
7	Carboplatin+Gemcitabine	**Alpelisib+Fulvestrant**	Phase1
8	NA	NA	NA
9	NA	NA	NA
10	Paclitaxel	**Alpelisib+Fulvestrant**	NA
11	NA	NA	NA
12	Carboplatin+Gemcitabine	Adriamycin	**Alpelisib+Fulvestrant**
13	Vinorelbine	**Alpelisib+Fulvestrant**	Eribulin

Table S1B- Detailed NGS test

Patient #	Test
1	N/A
2	CeGat
3	CE-IVD 22genes
4	Guardant360
5	N/A
6	FoundationCDx
7	FoundationCDx
8	BGI
9	Oncomine
10	FoundationCDx
11	FoundationCDx
12	VariantPlex Comprehensive Thyroid and Lung (CTL) panel (Invitae)
13	Oncomine

N/A – Not available.
